# Impaired Glucocorticoid Receptor Signaling Aggravates Lung Injury after Hemorrhagic Shock

**DOI:** 10.3390/cells11010112

**Published:** 2021-12-30

**Authors:** Jonathan M. Preuss, Ute Burret, Michael Gröger, Sandra Kress, Angelika Scheuerle, Peter Möller, Jan P. Tuckermann, Martin Wepler, Sabine Vettorazzi

**Affiliations:** 1 Institute of Comparative Molecular Endocrinology (CME), Ulm University, 89081 Ulm, Germany; jonathan.preuss@uni-ulm.de (J.M.P.); ute.burret@uni-ulm.de (U.B.); jan.tuckermann@uni-ulm.de (J.P.T.); 2 Institute for Anesthesiologic Pathophysiology and Process Engineering, Ulm University, 89081 Ulm, Germany; michael.groeger@uni-ulm.de (M.G.); sandra.weber@uni-ulm.de (S.K.); martin.wepler@uni-ulm.de (M.W.); 3 Institute of Pathology, University Hospital, 89081 Ulm, Germany; Angelika.Scheuerle@bkh-guenzburg.de (A.S.); peter.moeller@uniklinik-ulm.de (P.M.); 4 Department of Anesthesiology and Intensive Care Medicine, University Hospital, 89081 Ulm, Germany

**Keywords:** glucocorticoid receptor, homodimer, hemorrhagic shock, resuscitation

## Abstract

We previously showed that attenuated lung injury after hemorrhagic shock (HS) coincided with enhanced levels of the glucocorticoid (GC) receptor (GR) in lung tissue of swine. Here, we investigated the effects of impaired GR signaling on the lung during resuscitated HS using a dysfunctional GR mouse model (GR^dim/dim^). In a mouse intensive care unit, HS led to impaired lung mechanics and aggravated lung inflammation in GR^dim/dim^ mice compared to wildtype mice (GR^+/+^). After HS, high levels of the pro-inflammatory and pro-apoptotic transcription factor STAT1/pSTAT1 were found in lung samples from GR^dim/dim^ mice. Lungs of GR^dim/dim^ mice revealed apoptosis, most likely as consequence of reduced expression of the lung-protective *Angpt1* compared to GR^+/+^ after HS. RNA-sequencing revealed increased expression of pro-apoptotic and cytokine-signaling associated genes in lung tissue of GR^dim/dim^ mice. Furthermore, high levels of pro-inflammatory cytokines and iNOS were found in lungs of GR^dim/dim^ mice. Our results indicate impaired repression of STAT1/pSTAT1 due to dysfunctional GR signaling in GR^dim/dim^ mice, which leads to increased inflammation and apoptosis in the lungs. These data highlight the crucial role of functional GR signaling to attenuate HS-induced lung damage.

## 1. Introduction

Glucocorticoids (GCs) are frequently used to treat inflammation due to their anti-inflammatory effects mediated via the glucocorticoid receptor (GR) [1]. The ligand-bound GR as a transcription factor impacts gene expression of anti- and pro-inflammatory genes in different multimerization states. GR homodimer binds glucocorticoid-response elements (GRE) while monomeric GR either binds DNA half-sites (half GRE) or tethers transcription factors [2]. GR dimerization was shown to be responsible for the GC dependent induction of anti-inflammatory genes [3] such as *Tsc22d3* (GILZ) [4] and *Dusp1* (MKP-1, DUSP1) [5]. Monomeric GR represses inflammatory signaling pathways, e.g., by tethering pro-inflammatory transcription factors such as NFκB [6] or AP-1 [7]. We previously showed that functional GR is crucial for the resolution of systemic inflammation (endotoxemia) [8]. However, this mouse model was performed under non-intensive care conditions while patients usually receive intensive care management during systemic inflammation. Using a mouse model with affecting complete GR dimerization capacity induced by a point mutation in the DNA-binding domain (DBD) of the GR (GR^dim/dim^ mice [2,9]), we recently showed that impaired GR aggravates pulmonary dysfunction during endotoxemia under intensive care management [10]. Furthermore, in a large-animal model of arteriosclerotic swine undergoing hemorrhagic shock (HS) followed by resuscitation in an intensive care unit (ICU) setting, we found that sodium thiosulfate dependent attenuation of lung damage coincided with increased GR expression in the lungs [11]. This raised the question of the role of GR during HS-induced lung injury. Therefore, we investigated the effects of impaired GR in HS using the GR^dim/dim^ mouse model subjected to severe HS by withdrawal of ~30% blood volume for 1 h. Subsequently, mice were resuscitated in a mouse ICU (MICU) by support of fluids and norepinephrine, controlled body temperature and lung-protective ventilation as described before [10]. Here, we found in lung tissue of GR^dim/dim^ mice, enhanced levels of the transcription factor STAT1/pSTAT1, which possibly led to a reduced expression of angiopoietin-1 (*Angpt1*). *Angpt1* was shown to inhibit apoptosis after HS [12], and the reduced *Angpt1* expression coincided with an enhanced apoptosis of GR^dim/dim^ lung tissue after HS. Likewise, increased STAT1/pSTAT1 possibly enhanced the production of the pro-inflammatory cytokines and inducible nitrogen oxide synthase (iNOS) in lung tissue of GR^dim/dim^ mice. We here found that dysfunctional GR impaired lung compliance and aggravated HS-induced lung inflammation possibly via a newly proposed GR-STAT1-iNOS/*Angpt1* signaling pathway.

## 2. Materials and Methods

### 2.1. Animal Housing

The study was performed in adherence with the National Institutes of Health Guidelines on the Use of Laboratory Animals and the European Union “Directive 2010/63 EU on the protection of animals used for scientific purposes” and approved by the federal authorities for animal research of the Regierungspräsidium Tübingen, Baden-Wuerttemberg, Germany. GR^dim/dim^ mice (Nr3c1^tm3Gsc^ [9], background 129/SvEv × C57BL/6) and littermate control GR^+/+^ mice were kept in an animal facility at Ulm University. The mice were housed under standardized conditions (light/dark cycle of 12 h/12 h). For the experiments, mice were equally distributed in terms of age, body weight and sex. All animals were between 10 and 16 weeks old and weighed between 23 g and 34 g. In total, 7 GR^+/+^ mice (3 male, 4 female) and 9 GR^dim/dim^ mice (4 male, 5 female) were used. The HS experiments were done independently with one mouse per day.

### 2.2. Mouse Intensive Care Unit (MICU)

The in vivo experiments were performed in the MICU as described elsewhere [13]. In short, animals were anesthetized with sevoflurane inhalation (2.5% sevoflurane, Abbott, Wiesbaden, HE, Germany) and subsequently with intraperitoneal injection (i.p.) of ketamine (120 µg/g, Ketanest-S, Pfizer, New York City, NY, USA), midazolam (1.25 µg/g, Midazolam-ratiopharm, Ratiopharm, Ulm, BW, Germany) and fentanyl (0.25 µg/g, Fentanyl-hameln, Hameln Pharma Plus GmbH, Hameln, NI, Germany). Throughout the experiment, the animals received intra venous (i.v.) continuous infusion of 30 µg/g*h ketamine and 0.3 µg/g*h fentanyl to guarantee deep sedation. Catheters were installed into the jugular vein, the carotid artery, the femoral artery and the bladder. Body temperature was kept permanently at 37.0–37.5 °C via a closed-loop system [13]. Lung-protective ventilation was performed with a small animal ventilator (FlexiVent, SCIREQ, Montreal, QC, Canada) via trachestomy using FiO_2_ 21%, respiratory rate of 150/min, tidal volume of 6 mL/kg and a ratio of 1:2 for inspiratory to expiratory time. If necessary, ventilation was adjusted to maintain arterial PCO_2_ at 30–45 mmHg. Positive end-expiratory pressure (PEEP) was adjusted according to the Horovitz Index (PO_2_/FiO_2_ > 300 mmHg: PEEP = 3 cmH_2_O, PO_2_/FiO_2_ < 300 mmHg: PEEP = 5 cmH_2_O, PO_2_/FiO_2_ < 200 mmHg, PEEP = 8 cmH_2_O). Every hour, a recruitment maneuver was performed (18 cmH_2_O for 5 s) to avoid anesthesia or supine-position-induced atelectasis.

### 2.3. Hemorrhagic Shock (HS) Model

HS was induced by reduction of the mean arterial blood pressure (MAP) to 35 mmHg for 1 h via withdrawal of 30–40% of the total blood volume (20–30 µL/g) from the femoral artery. Coagulation of the withdrawn blood was prevented by addition of citrate. The blood was re-transfused after 1 h and mice were resuscitated for 4 h with infusion of 20 µL/g∗h colloids (Voluven^®^, Fresenius Kabi Deutschland GmbH, Bad Homburg, HE, Germany) and administration of norepinephrine (maximal infusion rate 1.5 µg/g∗h) to keep MAP > 55 mmHg. The mice were euthanized at the end of the resuscitation for sample collection.

### 2.4. Western Blot

Lung samples were taken at the end of the experiment and quick frozen on dry ice. After lysis in EDTA-free buffer, lungs were homogenized using a tissue homogenizer (Precellys^®^, PEQLAb Biotechnologie GmbH, VWR, Erlangen, BY, Germany) and total protein concentration was determined with Pierce^®^ BCA Protein Assay Kit (Thermo Scientific^TM^, ThermoFisher Scientific, Rockford, IL, USA). Proteins were separated using 10% SDS-PAGE and blotted on nitrocellulose membrane (Trans-Blot Turbo Transfer System, Bio-Rad, Feldkirchen, BY, Germany) with Bio-Rad Trans-Blot Turbo System. After blocking with 5% BSA for 1 h at room temperature, iNOS was detected with anti-mouse rabbit antibody (sc-651, Santa Cruz Biotechnology Inc., Heidelberg, BW, Germany), STAT1 was detected with anti-mouse rabbit antibody (9172, Cell Signaling Technology, Danvers, MA, USA), phosphorylated STAT1 (Tyr701, pSTAT1) was detected with anti-mouse rabbit antibody (Cell Signaling Technology, 9167) and osteopontin was detected with anti-mouse mouse antibody (LFMb-14, Santa Cruz Biotechnology Inc., Heidelberg, BW, Germany). All primary antibodies were diluted 1:500 in 5% BSA in TBST. Incubation with primary antibody was over night at 4 °C. As loading control, β-actin (A2228, Sigma-Aldrich, Saint Louis, MO, USA) or vinculin (sc-73614, Santa Cruz Biotechnology Inc., Heidelberg, BW, Germany) was detected using anti-mouse mouse antibody diluted 1:1000 in 5% BSA in TBST. Primary rabbit antibodies were detected with HRP-coupled secondary anti-rabbit goat antibody (65-6120, Invitrogen, Carlsbad, CA, USA) and primary mouse antibodies with anti-mouse rabbit HRP-coupled secondary antibody (P0161, Dako, Agilent, Santa Clara, CA, USA). For all washing and dilution steps, 0.1% TBST was used. Blots were developed using Immobilion Forte (WBLUF0500, Merck Millipore, Burlington, MA, USA) and ImageLab Software 5.2. Quantification was done by mean intensity determination using ImageJ 1.52a. Final arbitrary units were calculated using the ratio of the mean band intensity from the target protein to β-actin or to vinculin. Only samples of GR^dim/dim^ and GR^+/+^ run together on the same gel were used for analysis.

### 2.5. Bulk RNA-Sequencing of Lung Tissue

From three animals of each genotype (GR^+/+^: two female, one male, GR^dim/dim^: one female, two male) RNA was isolated from lung tissue samples obtained at the end of experiment. Bulk mRNA-sequencing was performed by Novogene (Novogene, Cambridge, UK). Expression data were analyzed with the DESeq2 package [14], principal component analysis (PCA) with the pcaExplorer package [15]. Data were visualized using the EnhancedVolcano package (Blighe, Rana and Lewis (2018)) and the ComplexHeatmap package [16]. For gene ontology (GO) analysis, PANTHER [17] and the generic GO term mapper of Princeton University was used.

### 2.6. Histology

Histological analysis hematoxylin and eosin stained lung sections were independently performed by two experienced pathologists (A.S. and P.M.) blinded for group assignment. Similar to previous studies [10,18], analyzed criteria comprised thickening of alveolar membranes, dystelectasis, emphysema, inflammatory cell (lymphocytes and macrophages) infiltration and inflammatory alterations. Alveolar membrane thickening was scored from 1 (marginally enlarged) to 4 (maximally enlarged) in the analyzed specimens. Dystelectasis was scored according to appearance of dystelectasis (+1), fresh bleeding (+5) and presence of oedema (+15), which were added to the total score presented. Appearance of emphysema was scored from 1 (mild) to 3 (significant). Intensity of presence of lymphocytes was scored from 1 (low) to 4 (profound). Macrophages were counted according to their appearance in 10 high-power field (HPF) and inflammatory alteration was scored from 1 (minimal) to 5 (maximal alteration) in the analyzed specimen. Per mouse, two lung slices as technical replicates were analyzed.

### 2.7. Immunohistochemical (IHC) Detection of Lung Albumin Level as Indicator for Vascular Permeability

Albumin was detected by IHC on paraffin fixated lung slices from 9 GR^dim/dim^ mice and 6 GR^+/+^ mice. Per mouse, two lung slices were analyzed as technical replicates. After deparaffination and rehydration, for antigen retrieval, slides were incubated 25 min in hot 1× citrate buffer pH 6.0 (C9999, Sigma-Aldrich, Saint Louis, MO, USA). Slices were blocked with blocking reagent (Merck Millipore, 20773) for 2 h at 4 °C, followed by albumin detection using anti-mouse rabbit antibody (Proteintech, 16475-1-AP) diluted 1:150 and incubated over night at 4 °C. The next day, endogenous peroxidases were deactivated by 15 min incubation in 0.3% H_2_O_2_ and endogenous avidin and biotin was saturated by incubation with avidin/biotin blocking (SP-2001, Vector Laboratories Inc., Burlingame, CA, USA) 15 min each. Primary antibody was detected with biotinylated anti-rabbit goat antibody (BA-100, Vector Laboratories Inc., Burlingame, CA, USA) diluted 1:800 and incubated 2 h at 4 °C followed by incubation with 1:300 diluted streptavidin-coupled peroxidase (Vector Laboratories, Inc., Burlingame, CA, USA) for 1 h at 4 °C. Subsequent incubation with 3,3′-diaminobenizidin (DAB, SK-4105, Vector Laboratories Inc., Burlingame, CA, USA) was done for 45 s per slice. DAB incubation was terminated by washing with running tap water. For counter-staining, slices were incubated with hematoxylin (H-3401, BIOZOL, Eching, BY, Germany) for 5 min followed by staining differentiation with 2% glacial acetic, solution A (25% NH_4_OH diluted 1:56 in 70% ethanol) and solution B (25 NH_4_OH diluted 1:250 in tap water). After dehydration, slices were mounted (Eukitt mounting medium, 03989, Sigma-Aldrich, Saint Louis, MO, USA) and pictures were taken with 4× magnification. For analysis, from each technical replicate, one picture was taken and DAB stained area was determined after color deconvolution and threshold adjustment in ImageJ. For quantification, the mean of the two technical replicates per mouse were used.

### 2.8. Assessment of Relative mRNA Expression with Quantitative real Time Polymerase Chain Reaction (qRT-PCR)

Lung samples of GR^dim/dim^ mice and GR^+/+^ mice were prepared for qRT-PCR by homogenization in TRIzol (15596026, Invitrogen, Carlsbad, CA, USA) with tissue homogenizer (Precellys^®^) according to the manufacturer’s protocol. RNA was transcribed to cDNA using SuperScript^TM^ reverse transcriptase (18080044, Invitrogen, Carlsbad, CA, USA). qRT-PCR was performed with Platinum SYBR Green (11733038, Invitrogen, Carlsbad, CA, USA) with ViiA^TM^ 7 Real-Time PCR System (Life technologies, Carlsbad, CA, USA) and QuantStudio Real-Time PCR software. mRNA expression of *Spp1* (forward primer: 5′ CAGTGATTTGCTTTTGCCTGT 3′, reverse primer: 5′ CTCCTCTGAGCTGCCAGAAT 3′) and *Angpt1* was quantified with ΔΔCT method by normalization to *β-actin* (forward primer: 5′ GCACCAGGGTGTGATGGTG 3′, reverse primer: 5‘ CCAGATCTTCTCCATGTCGTCC 3′) and *ribosomal protein large* (*RPL*) (forward primer: 5′ CCTGCTGCTCTCAAGGTT 3′, reverse primer: 5′ TGGCTGTCACTGCCTGGTACTT 3′). In total, samples of all 9 GR^dim/dim^ were analyzed and samples of 6 GR^+/+^ mice were analyzed due to restriction of material from one GR^+/+^ mice. Every sample was measured as triplicate and the mean of all three technical replicates was used for quantification.

### 2.9. TUNEL Assay for Apoptosis Measurement

Lung sections of all 9 GR^dim/dim^ and 7 GR^+/+^ mice were assayed for apoptosis with in situ cell death detection kit (11684795910, Roche, Mannheim, BW, Germany) according to manufacturer’s protocol. In short, paraffin lung sections were deparaffined and dehydrated prior to permeabilization. DNA strand breaks were detected as marker for apoptosis by labeling with fluorescein-coupled dUTP. The sections were mounted in DAPI-enriched mounting medium (VEC-H-1200, Vectashield, BIOZOL, Eching, BY, Germany). Fluorescein fluorescence intensity of 40× pictures was measured only in the nuclei identified by DAPI staining using CellProfiler [19]. Of each lung slice from the 9 GR^dim/dim^ and 7 GR^+/+^ mice, three pictures from distinct areas of the lung slice were analyzed. The mean nuclear fluorescent signal of all three pictures were used for quantification.

### 2.10. Bio-Plex Based Measurement of Cytokines and Chemokines

Cytokines and chemokines were determined in bronchoalveolar lavage (BAL) samples using a Bio-Plex Pro Mouse Cytokine 23-plex Assay (Group I) (Bio-Rad, Feldkirchen, BY, Germany) as described elsewhere [20]. For readout, a Bio-Plex 200 machine (Bio-Rad, Feldkirchen, BY, Germany) was used with respective analysis software (Bio-Plex Manager TM 6.1, Bio-Rad, Hercules, CA, USA).

### 2.11. Statistics and Graph Visualization

Statistical analysis and graphs were made with GraphPad Prism 8.0.1 (GraphPad Software, La Jolla, CA, USA). For statistics, unpaired Mann–Whitney was used. Significant outliers were detected using GraphPad Grubbs test for outliers and removed from the analysis. Data are presented by mean and standard error of the mean (SEM). Asterisk annotation was used according to * *p* < 0.05, ** *p* < 0.01, *** *p* < 0.001.

## 3. Results

### 3.1. Functional GR Is Crucial for Lung Mechanics after Hemorrhagic Shock

**Figure 1:**
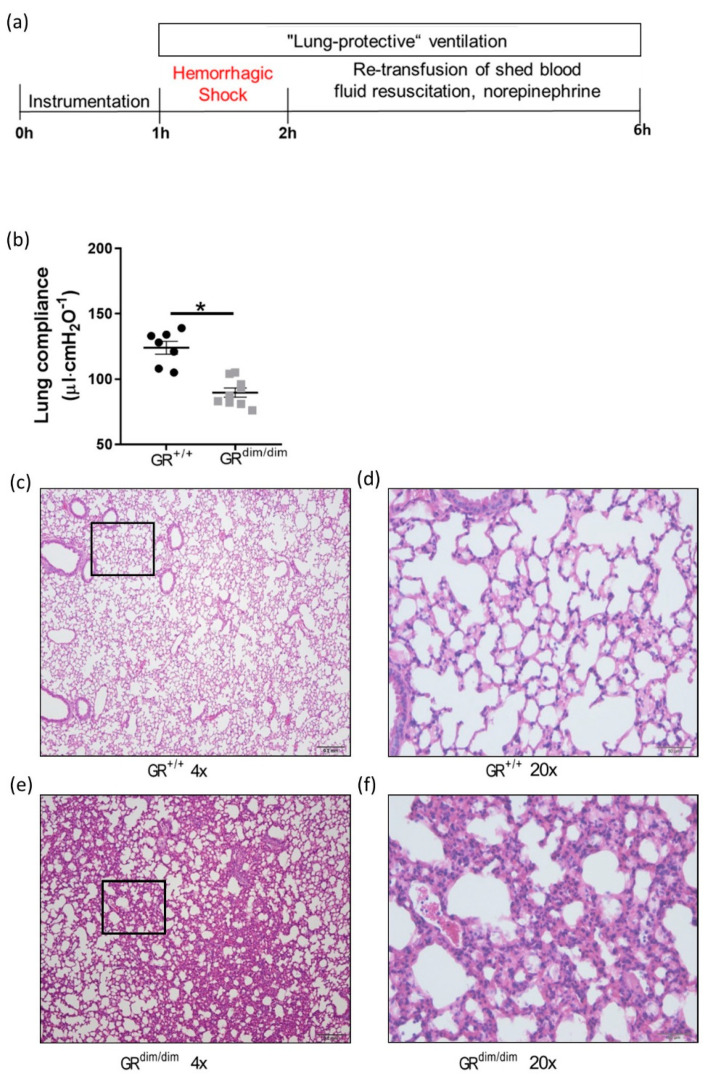
GR^dim/dim^ mice show pulmonary impairment after resuscitation from HS. (**a**) After instrumentation, all animals underwent HS followed by 4 h of resuscitation in MICU. After in total 6 h, the mice were euthanized for sample collection. (**b**) After HS, GR^dim/dim^ showed a reduced lung compliance at the end of the experiment as marker for impaired lung mechanics compared to GR^+/+^ after HS. (**c**) Exemplary hematoxylin and eosin (HE) stained lung section (4× magnification) from GR^+/+^ mouse with indicated area of (**d**) 20× magnification. (**e**) HE stained lung section of GR^dim/dim^ mouse (4× magnification) with indicated area of (**f**) 20× magnification. Quantifications are shown as mean with standard error of the mean (SEM). Significance was analyzed with unpaired Mann-Whitney and indicated according to * *p* < 0.05.

The consequences of impaired GR signaling on the lungs after HS were examined in a mouse intensive care unit (MICU). After instrumentation for thoracic lung-protective ventilation and catheters, GR^dim/dim^ and GR^+/+^ underwent severe [21] HS for 1 h with a reduced mean arterial blood pressure (MAP) of 35 mmHg by withdrawal of 30–40% arterial blood. Afterwards, the shed blood was re-transfused and mice were resuscitated for 4 h with support of fluids and norepinephrine (Figure 1a). After HS, GR^dim/dim^ mice had a significantly lower lung compliance compared to GR^+/+^ (Figure 1b). However, immunohistochemical investigation of albumin as marker for vascular permeability revealed no differences between lung sections from GR^dim/dim^ and GR^+/+^ mice (Supplementary Figure S1a–g). For further investigation, we examined pathological impairments of lung sections via histological analysis (Figure 1c–f). Lungs were scored according to their damage as seen by alveolar membrane thickening, dystelectasis, emphysema, the appearance of lymphocytes and macrophages, and inflammatory alteration. The lungs of GR^dim/dim^ mice showed a trend towards thicker alveolar membranes compared to lungs of GR^+/+^ after HS (Table 1). These findings coincided with tendencies towards blood gas imbalances of GR^dim/dim^ mice as seen by trends of increased arterial partial carbon dioxide pressure (PCO_2_, Supplementary Figure S1h) and decreased Horovitz Index (arterial partial pressure of oxygen divided by the fraction of inspiratory oxygen concentration, Supplementary Figure S1i). These lung impairments may have been the reason why only 77.78% of the GR^dim/dim^ mice survived while 100% of the GR^+/+^ mice survived until the end of the experiment (Supplementary Figure S1j).

### 3.2. RNA-Sequencing of Lung Tissue Samples from GR^dim/dim^ and GR^+/+^ Mice

**Figure 2:**
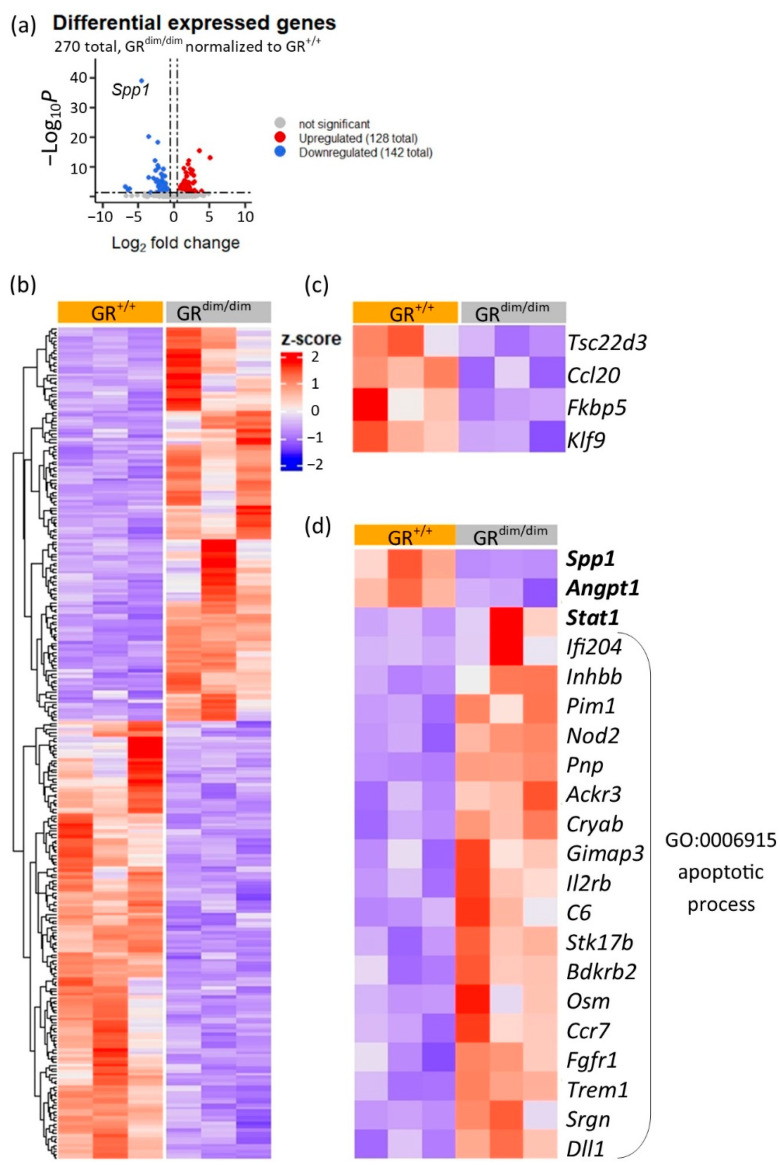
Bulk RNA-sequencing of lung samples from GR^dim/dim^ and GR^+/+^ mice after HS with subsequent resuscitation. (**a**) Up- and downregulated DEGs (thresholds: *p*-adjusted value < 0.05, fold change > 0.5) plotted by *p*-value (−Log_10_*P*) and fold change (Log_2_ fold change). (**b**) DEGs with a FDR < 0.05 z-score sorted for up- and downregulation relative to the expression level in GR^+/+^ lung tissue. (**c**) Heatmap of selected GR target genes showing impaired expression of GR target genes in lung tissue from GR^dim/dim^ mice. (**d**) Relative expression of *Spp1*, *Angpt1*, *Stat1* and genes upregulated in GR^dim/dim^ annotated with GO term “apoptotic processes”.

In order to assess potentially differential regulated genes that contributed to the impaired lung mechanics in GR^dim/dim^ mice after HS, lung tissue from GR^dim/dim^ and GR^+/+^ mice was further investigated by RNA-sequencing (three samples each). Principal component analysis (PCA) (Supplementary Figure S2a) and Euclidean distance clustering (Supplementary Figure S2b) showed that the gene expression profile differed more between genotypes than between samples of the same genotype. RNA-sequencing revealed 270 genes that exceeded the threshold for significantly (*p* < 0.05) differentially expressed genes (DEGs) between GR^dim/dim^ and GR^+/+^ lung tissue samples (Figure 2a). Normalization of the DEGs to their expression profile in GR^+/+^ lungs resulted in 128 upregulated and 142 downregulated genes in lung samples from GR^dim/dim^ mice (Figure 2a). Relative expression (z-score) of all DEGs between the lung samples of GR^dim/dim^ and GR^+/+^ mice is displayed as a heatmap in Figure 2b. The complete list of all DEGs with *p*-values and log_2_ fold change can be found in Supplementary Table S1 and at GSE192414. Impaired GR signaling in GR^dim/dim^ lungs was verified by reduced expression of GR target genes such as *Tsc22d3*, *Ccl20*, *Fkbp5* [4] and *Klf9* [22] (Figure 2c). The RNA expression profiling showed that dysfunctional GR heavily impacted gene expression in the lungs of GR^dim/dim^ mice after HS.

### 3.3. Enhanced Apoptotic Processes in GR^dim/dim^ after HS

We further investigated the DGEs and found that the most significant downregulated gene in lung samples from GR^dim/dim^ compared to GR^+/+^ mice after HS was *Spp1* (Figure 2a). The significantly reduced expression of *Spp1* in lungs of GR^dim/dim^ mice could be verified by qRT-PCR (Supplementary Figure S3a). Trends of reduced levels of the gene product of *Spp1*, osteopontin (OPN), were found on the protein level in the same lung tissue samples of GR^dim/dim^ mice that were used for RNA-sequencing (Supplementary Figure S3b) but not in the complete cohort of GR^dim/dim^ and GR^+/+^ mice after HS (Supplementary Figure S3c).

**Figure 3:**
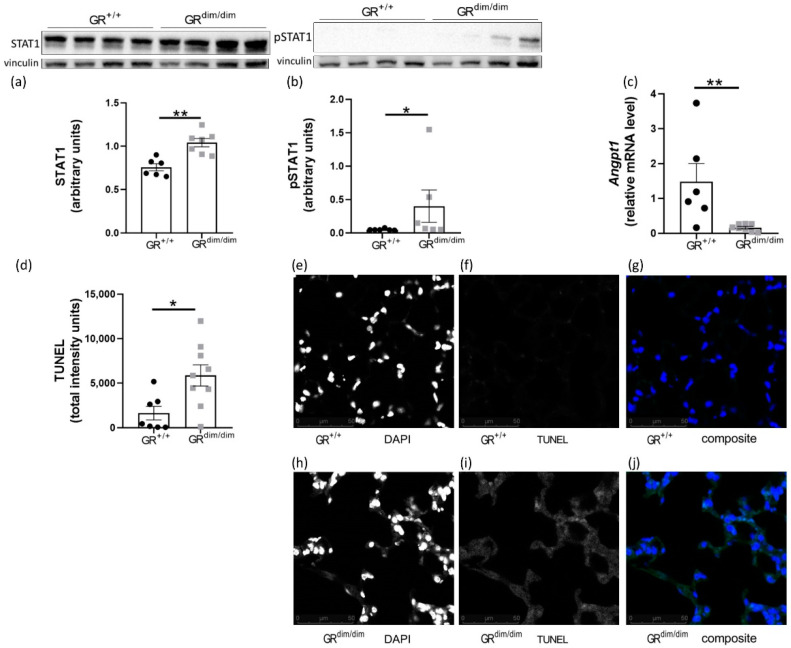
Increased apoptotic signaling in lungs from GR^dim/dim^ mice after HS. In lung tissue samples from GR^dim/dim^ mice, significantly (**a**) higher protein level of STAT1, (**b**) higher protein level of pSTAT1 and (**c**) reduced *Angpt1* expression was found compared to lung tissue of GR^+/+^ mice after HS. (**d**) Lung sections of GR^dim/dim^ mice showed stronger TUNEL staining indicating increased apoptosis compared to lung sections of GR^+/+^ after HS. Representative pictures at 40× magnification are shown for GR^+/+^ lung sections stained with (**e**) DAPI for detection of nuclei and (**f**) TUNEL (flourescein-dUTP) for detection of apoptosis and (**g**) both (composite). Representative pictures at 40× magnification are shown for GR^dim/dim^ lung sections stained with (**h**) DAPI, (**i**) TUNEL and (**j**) both. Only nuclear TUNEL signal was used for quantification. Data are shown as mean with SEM. Significance was analyzed with unpaired Mann-Whitney and indicated according to * *p* < 0.05, ** *p* < 0.01.

Interestingly, in lung tissue of GR^dim/dim^ mice, we found that STAT1 was significantly upregulated on RNA expression (Figure 2d) as well as protein level (Figure 3a) compared to GR^+/+^ after HS. STAT1 is a transcription factor, which impacts gene expression upon being phosphorylated (pSTAT1) [23]. In line with the increased STAT1 levels, significantly higher pSTAT1 levels were found in lung tissue of GR^dim/dim^ mice compared to GR^+/+^ after HS (Figure 3b). In endothelial cells, increase of pSTAT1 coincided with reduced expression of the inhibitor of apoptosis angiopoietin-1, *Angpt1* [24,25]. In lung tissue from GR^dim/dim^ mice, the increased pSTAT1 levels were accompanied with reduced expression of *Angpt1* compared to GR^+/+^ mice after HS as seen by RNA-sequencing (Figure 2d) and qRT-PCR (Figure 3c). *Angpt1* was shown to improve endothelial integrity after HS by inhibition of apoptotic signaling [12,25]. In line with reduced expression of *Angpt1*, 19 upregulated genes in lung tissue from GR^dim/dim^ mice after HS are annotated with the gene ontology (GO) term “apoptotic process” (GO ID GO:0006915, Figure 2d). For verification, we assayed apoptosis by fluorescent TUNEL staining to detect DNA fragmentation as marker for apoptosis. Significantly more TUNEL staining was detected in lung sections from GR^dim/dim^ mice compared to GR^+/+^ after HS (Figure 3d–j).

### 3.4. Enhanced Lung Inflammation in GR^dim/dim^ Mice after HS

**Figure 4:**
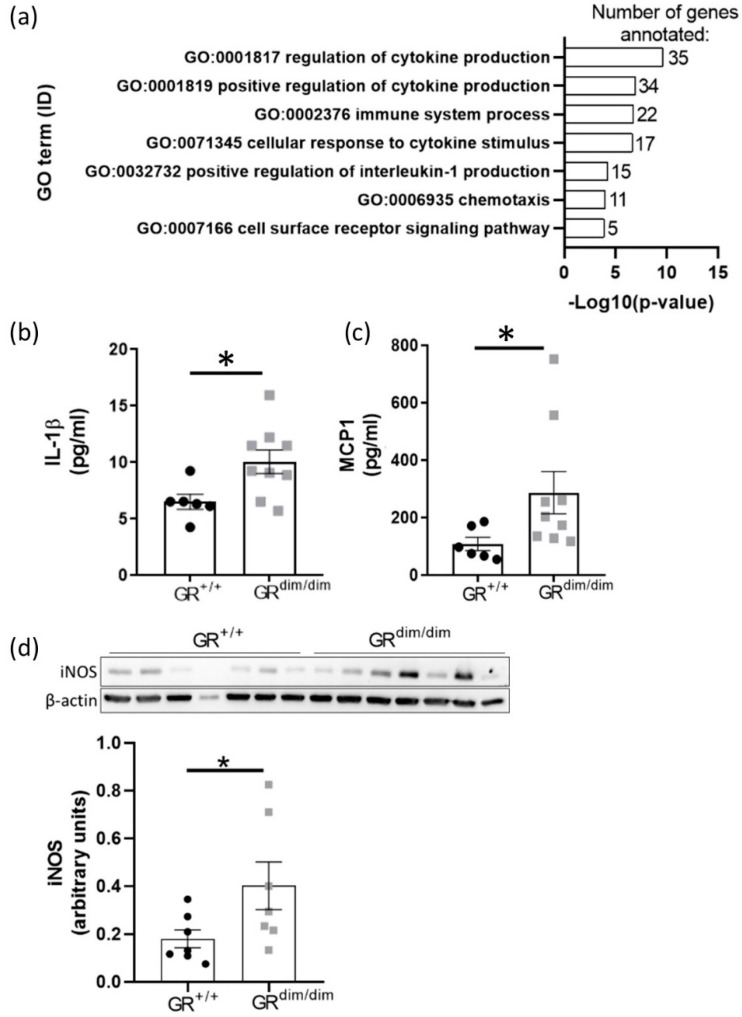
Enhanced cytokine and chemokine signaling in GR^dim/dim^ and GR^+/+^ mice after HS. (**a**) Gene ontology (GO) mapping of upregulated genes in lung samples from GR^dim/dim^ mice with indication of the gene number per GO term in the bars. The *X*-axis shows the significance for annotation with the respective GO term. (**b**) IL-1β and (**c**) MCP1 concentration in BAL samples obtained at the end of the experiment were increased in GR^dim/dim^ compared to GR^+/+^ mice. (**d**) GR^dim/dim^ mice had higher iNOS protein level in lung samples than GR^+/+^ mice after HS. Significance was analyzed with unpaired Mann-Whitney and indicated according to * *p* < 0.05.

Interestingly, we found several genes upregulated in the lungs of GR^dim/dim^ mice involved in the production and response to interleukin-1β (IL-1β) such as *Nod2* (processing of pro-IL-1β [26]) and *Ifi204* (mediator of toll-like receptor 4 (TLR4)-induced IL-1β production [27,28]) (Figure 2d). Furthermore, GO term mapping revealed that many of the 128 upregulated genes in lung tissue of GR^dim/dim^ mice were annotated with terms of cytokine and chemokine signaling (Figure 4a). To verify the RNA-sequencing results, we measured cytokines and chemokines in bronchoalveolar lavage (BAL) samples of GR^dim/dim^ and GR^+/+^ mice after HS. GR^dim/dim^ mice had elevated levels of pro-inflammatory cytokines including IL-1β (Figure 4b) and chemokines such as MCP1 (enhancer of infiltration of monocytes) (Figure 4c) in BAL samples (Supplementary Table S2). Furthermore, we found increased iNOS levels in the lungs of GR^dim/dim^ mice compared to GR^+/+^ after HS. These data show that impaired GR function aggravates HS-induced lung inflammation.

## 4. Discussion

### 4.1. Functional GR Is Crucial for Lung Health in Resuscitated Shock

We previously showed that attenuation of HS-induced lung injury coincided with increased GR levels in lung tissue of swine [11], indicating lung-protective effects mediated by the GR during HS. The aim of the present study was to investigate the effects of dysfunctional GR signaling on the lungs during resuscitated HS. We reported earlier on the central role of intact GR signaling LPS-induced acute lung injury [8], TNF-α-induced systemic inflammation [5] and cecal ligation, and puncture-induced sepsis [29]. However, these studies investigated the role of the GR dimer in non-clinical settings without resuscitation. In our recent studies, we showed that impaired GR signaling aggravated pulmonary dysfunction in resuscitated endotoxemia [10,30] in a translational mouse intensive care setup [31]. Using the same MICU setup for resuscitation, we here showed that impaired GR function (GR^dim/dim^) led to a reduced lung compliance after HS (Figure 1b). The same phenotype was found in our previous study on endotoxemia in GR^dim/dim^ mice [10]. Therefore, it can be speculated that intact GR function plays a crucial role for lung function during resuscitated endotoxic shock as well as hemorrhagic shock.

### 4.2. Full Funtional GR Is Necessary for Repression of HS-Induced STAT1/pSTAT1

The reduced lung compliance of GR^dim/dim^ mice after HS (Figure 1b) coincided with increased STAT1 and pSTAT1 levels in the lungs of GR^dim/dim^ mice compared to GR^+/+^ mice after HS. HS is known to increase STAT1 and pSTAT1 levels in the lungs [32] and increased levels of STAT1 and pSTAT1 were found in intestinal epithelial cells (IECs) of GR^dim/dim^ mice after TNF-α challenge [33]. Repression of STAT1 transcription by GR was proposed to be the consequence of GR binding to IR negative glucocorticoid response elements (IR nGREs) [34]. In fact, Ballegeer et al. showed that the increased STAT1 levels in IECs from GR^dim/dim^ mice were the consequence of absent GR binding to IR nGREs of *Stat1* [34]. Accordingly, we hypothesize that the increased STAT1 and pSTAT1 levels in lungs of GR^dim/dim^ mice compared to GR^+/+^ mice after HS were the consequence of impaired GR dependent repression of *Stat1* transcription.

### 4.3. Increased Apoptosis in Lungs of GR^dim/dim^ Mice after HS

Activation of the JAK2/STAT1 pathway is known to drive inflammatory processes and cell death [35]. The elevated STAT1/pSTAT1 levels in lung tissue of GR^dim/dim^ mice (Figure 3a,b) coincided with increased expression of genes associated with apoptotic processes (Figure 2d) and reduced expression of *Angpt1* (Figure 2d and Figure 3c). *Angpt1* improves endothelial integrity by the inhibition of apoptotic signaling [12]. Reduced expression of *Angpt1* was reported in human endothelial cells to coincide with increased pSTAT1 [24]. In line with the reduced expression of *Angpt1*, we found increased apoptosis in lung tissue of GR^dim/dim^ mice compared to GR^+/+^ mice after HS (Figure 3d). Enhanced apoptosis accompanied by elevated pSTAT1 levels were reported in IECs of GR^dim/dim^ mice [33]. We therefore propose that in lungs of GR^dim/dim^ mice, the impaired GR failed to repress *Stat1* expression, which led to reduced expression of *Angpt1* and thereby to enhanced apoptosis.

### 4.4. Impaired GR Enhances HS-Induced Lung Inflammation

Overexpression of *Angpt1* was shown to protect mice from LPS-induced acute lung injury by reduced expression of pro-inflammatory cytokines in the BAL [36] and decreased lung damage via inter alia reduced swelling of alveolar walls [37]. Together with the reduced expression of *Angpt1* in lung tissue of GR^dim/dim^ mice, we found increased expression of genes associated with cytokine signaling in the lungs (Figure 4a) and increased pro-inflammatory cytokine levels in the BAL of GR^dim/dim^ mice compared to GR^+/+^ after HS (Figure 4b,c, Supplementary Table S2). Moreover, we found enhanced levels of the pro-inflammatory iNOS in lung tissue of GR^dim/dim^ mice compared to GR^+/+^ after HS (Figure 4d). HS was shown to increase iNOS in the lungs [38] and kidneys [39] of rats. In the kidneys, HS-induced iNOS coincided with increased pSTAT1 levels [39]. Increased iNOS levels in the lungs are associated with aggravated lung damage after HS [38,40] and during lung inflammation, reduced lung compliance was shown to depend on the pro-inflammatory protein iNOS [41]. Similar to the proposed regulation of *Angpt1*, we hypothesize that the impaired GR-dependent suppression of STAT1 led to enhanced levels of iNOS and pro-inflammatory cytokines and, therefore, to increased HS-induced lung inflammation in GR^dim/dim^ mice compared to GR^+/+^ mice.

## 5. Conclusions

**Figure 5:**
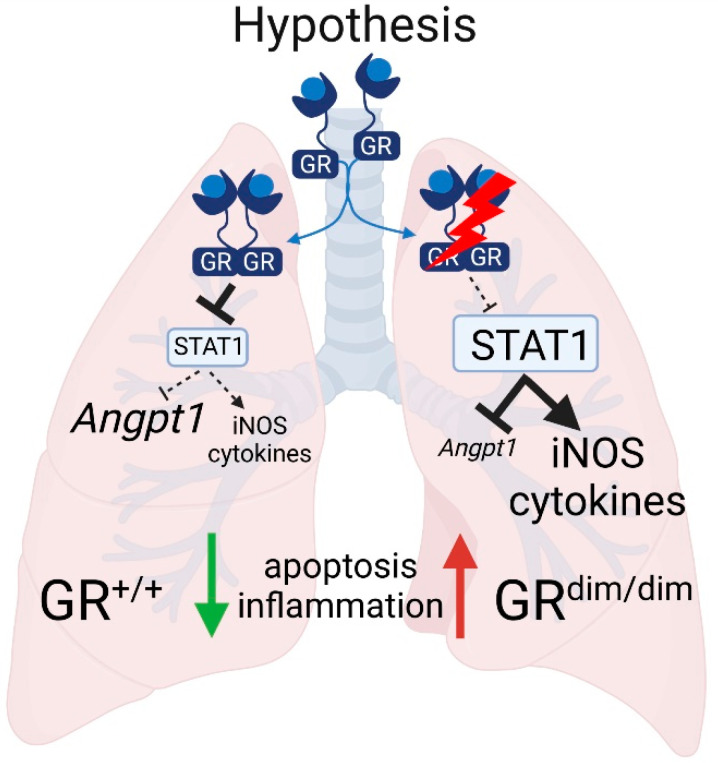
In resuscitated HS, the GC bound GR dimer may impact lung injury via repression of STAT1-dependent expression of the pro-inflammatory iNOS and enhanced expression of the lung-protective *Angpt1*. The figure was created with biorender.com (accessed on 23 December 2021).

We showed that impaired GR increased apoptosis and inflammation in the lungs and reduced the lung compliance after resuscitated HS in mice. Our findings suggest that functional GR signaling plays a key role in attenuation of lung injury during resuscitated HS via GR-STAT1-iNOS/*Angpt1* signaling (Figure 5). These findings support and extend our previous hypothesis that increased GR levels mediate lung-protective effects after HS [11]. The recognition of GR-dependent repression of STAT1 signaling as central mechanism for the attenuation of lung injury after HS reveals new potential targets for medical interventions of HS-induced lung injury.

## Figures and Tables

**Table 1:** Quantification of the pathological grading of lung sections from GR^+/+^ (*n* = 7) and GR^dim/dim^ (*n* = 9) mice after HS with subsequent resuscitation. Data are shown as mean (SEM).

Parameters	GR^+/+^	GR^dim/dim^	*p*-Value
Alveolar membrane thickening	1.0 (0)	1.3 (0.15)	0.0799
Dystelectasis	10 (2.5)	6.4 (2.1)	0.3100
Emphysema	1.3 (0.18)	1.8 (0.22)	0.2290
Lymphocytes	1.0 (0)	1.1 (0.16)	0.8981
Macrophages	8.5 (0.96)	7.5 (0.53)	0.3583
Inflammatory alteration	1.4 (0.18)	1.7 (0.24)	0.3245

## Data Availability

Data is uploaded at GEO:GSE192414.

## Supplementary Materials

The following are available online at https://www.mdpi.com/article/10.3390/cells11010112/s1, Figure S1: Albumin appearance on lung sections from GR^dim/dim^ and GR^+/+^ mice, PCO_2_, Horovitz-Index and survival rates of GR^dim/dim^ and GR^+/+^ mice after HS with subsequent resuscitation. Figure S2: Quality assessment bulk RNA-sequencing. Figure S3: qRT-PCR and western blot analysis of osteopontin in lung samples of GR^dim/dim^ and GR^+/+^ mice after HS. Table S1: Overview of all DGE found by RNA-sequencing of lung tissue samples from GR^dim/dim^ and GR^+/+^. Table S2: BAL cytokine levels of GR^dim/dim^ and GR^+/+^ mice after HS.
