# Supplementary material for: Impaired Glucocorticoid Receptor Signaling Aggravates Lung Injury after Hemorrhagic Shock
Source: Cells. 2021 Dec 30;11(1):112. doi: 10.3390/cells11010112 (PMC8750862; doi:10.3390/cells11010112)
Supplement: Supplementary file 1 [file cells-11-00112-s001.zip › cells-1505937-supplementary.pdf]

cells

MDPI

Article

Impaired Glucocorticoid Receptor Signaling Aggravates Lung Injury after Hemorrhagic Shock

Jonathan M. Preuss [1], Ute Burret [1], Michael Gröger [2], Sandra Kress [2], Angelika Scheuerle [3], Peter Möller [3], Jan P. Tuckermann [1], Martin Wepler [2,4,†], Sabine Vettorazzi [1,*,†]

1 Institute of Comparative Molecular Endocrinology (CME), Ulm University, Ulm, Germany
2 Institute for Anesthesiologic Pathophysiology and Process Engineering, Ulm University, Ulm, Germany
3 Institute of Pathology, University Hospital, Ulm, Germany
4 Department of Anesthesiology and Intensive Care Medicine, University Hospital, Ulm, Germany
* Correspondence: sabine.vettorazzi@uni-ulm.de
† These authors have contributed equally to this work.

Supplementaries

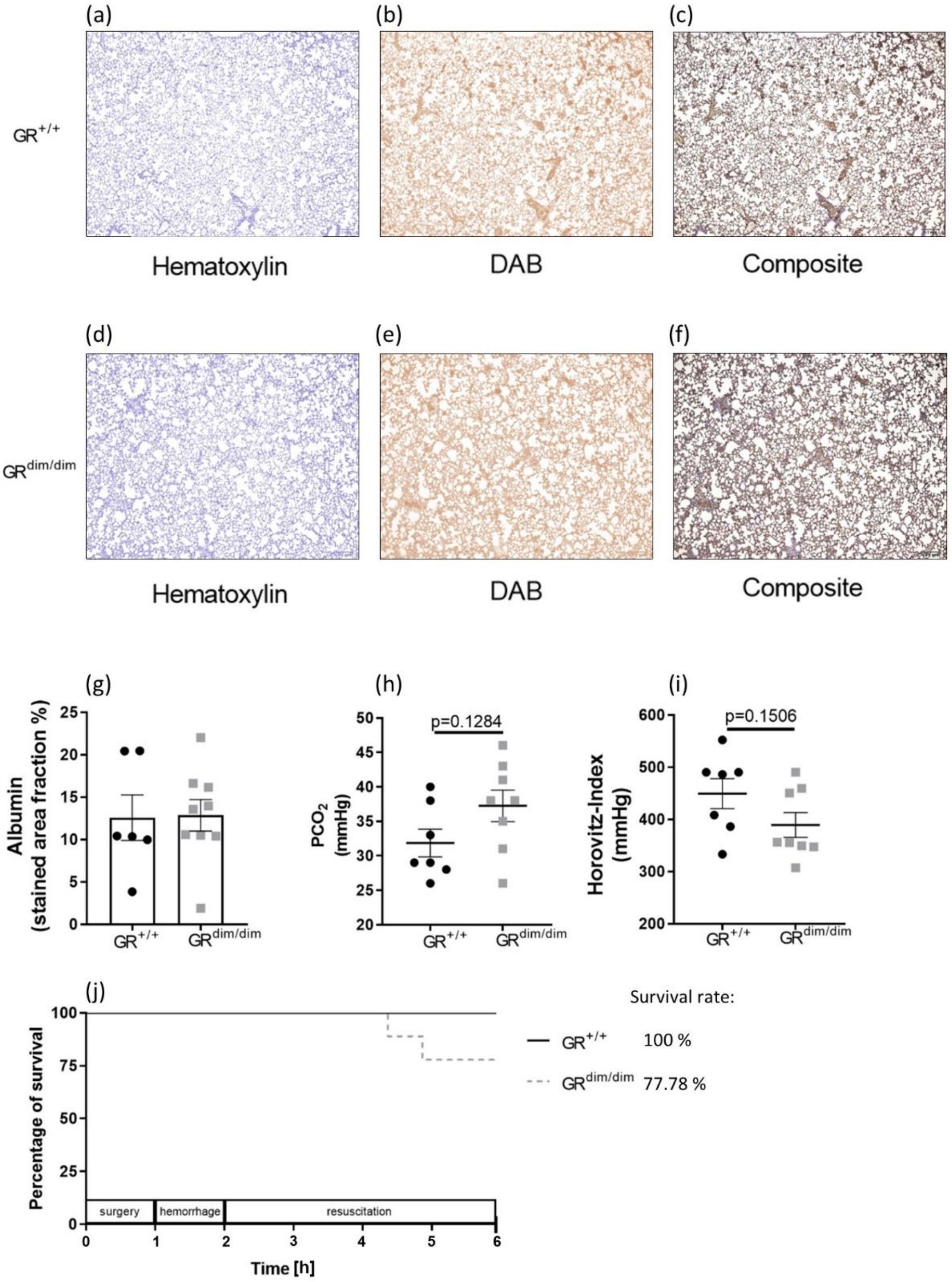

Figure S1: Vascular permeability, blood gas analysis and survival of $GR^{dim/dim}$ mice and $GR^{+/+}$ mice after HS in MICU. (**a**) Representative hematoxylin counter-stained lung slice of $GR^{+/+}$ mouse. (**b**) DAB signal for albumin detection on lung slice from $GR^{+/+}$ mouse. (**c**) Overlay of hematoxylin and DAB staining as composite of $GR^{+/+}$ mouse. Below, representative pictures of lung slice from a $GR^{dim/dim}$ mouse with (**d**) hematoxylin staining, (**e**) DAB staining and (**f**) composite. All pictures were taken with 4× magnification. (**g**) Stained area fraction of albumin was not different between lung slices of $GR^{dim/dim}$ mice and $GR^{+/+}$ mice. (**h**) HS led to trends of enhanced arterial partial carbon dioxide pressure (PCO_2) in $GR^{dim/dim}$ mice compared to $GR^{+/+}$. (**i**) $GR^{dim/dim}$ mice showed tendecies of a reduced Horovitz-Index (ratio of inhaled oxygen fraction to arterial partial oxygen pressure) after HS compared to $GR^{+/+}$. (**j**) 77.78 % of $GR^{dim/dim}$ mice survived at the end of the experiment while all $GR^{+/+}$ mice survived. Significance was analyzed with unpaired Mann-Whitney and indicated according to * $p < 0.05$, ** $p < 0.01$, *** $p < 0.001$.

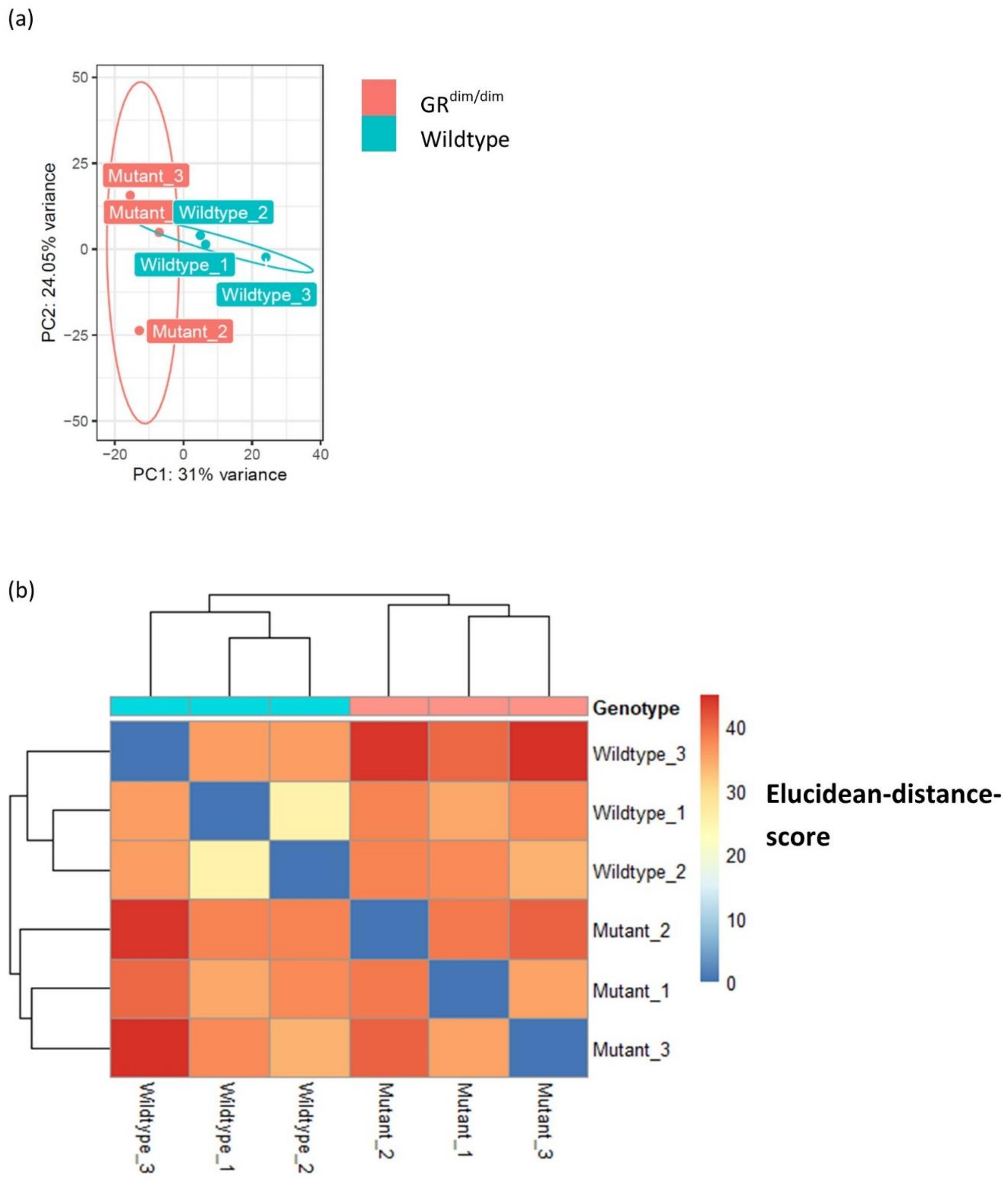

Figure S2: Quality assessment of RNA-sequencing results. (**a**) PCA blot showing clustering of the samples according to the genotype for their variance (PCA1 and PCA2). (**b**) Heatmap of elucidean-distance-clustering in a sample-to-sample comparison indicating least (blue) to most (red) differences in gene expression profile. Dendograms cluster the samples according to their similarities.

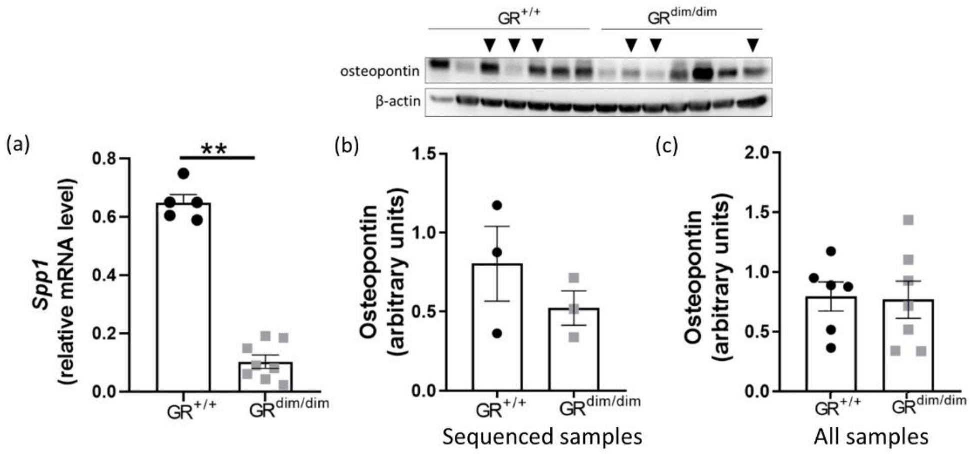

Figure S3: $GR^{dim/dim}$ mice showed reduced RNA but not protein level of osteopontin in the lung after HS. (**a**) qRT-PCR showed significantly reduced *Spp1* expression in lung samples of $GR^{dim/dim}$ mice compared to lung samples of $GR^{+/+}$ mice after HS. (**b**) Analysis of the lung tissue samples that were analyzed by RNA-sequencing (indicated by arrow heads in the blot) showed trends towards reduced protein level of osteopontin (OPN), the geneproduct of *Spp1*, in $GR^{dim/dim}$ lungs compared to $GR^{+/+}$ after HS. (**c**) Western blot of lungs samples from all $GR^{dim/dim}$ and $GR^{+/+}$ mice after HS showed no genotype dependent differences of OPN. Data points shown in (**b**) are included in (**c**). Significance was analyzed with unpaired Mann-Whitney and indicated according to * $p < 0.05$, ** $p < 0.01$, *** $p < 0.001$.

Table S2. Concentration of cytokines in BAL of $GR^{+/+}$ (n=6-7) and $GR^{dim/dim}$ (n=8-9) mice after hemorrhagic shock with subsequent resuscitation (4 h) at the end of experiment. *p<0.05 vs. $GR^{+/+}$ calculated with Mann-Whitney t-test. Data is shown as mean (SEM).

**Cytokine in BAL [pg/ml]**	**$GR^{+/+}$**	**$GR^{dim/dim}$**
Interleukine-1 alpha (IL-1 α)	9.7 (0.47)	9.3 (0.70)
Interleukine-2 (IL-2)	7.7 (0.64)	7.5 (0.77)
Interleukine-3 (IL-3)	1.0 (0.11)	1.6 (0.18)
Interleukine-4 (IL-4)	1.8 (0.22)	1.5 (0.079)
Interleukine-5 (IL-5)	6.1 (0.95)	7.7 (0.93)
Interleukine-6 (IL-6)	544 (105)	777 (98)
Interleukine-9 (IL-9)	6.9 (1.0)	9.8 (0.52)
Interleukine-10 (IL-10)	4.0 (0.53)	4.7 (0.68)
Interleukine-12 p40 (IL-12 p40)	16 (1.6)	13 (1.0)
Interleukine-12 p70 (IL-12 p70)	48 (5.0)	54 (3.8)
Interleukine-13 (IL-13)	20 (0.73)	**28 (3.0)***
Interleukine-17 (IL-17)	3.6 (0.16)	4.3 (0.29)
Eotaxin	93 (13)	144 (24)
Granulocyte Colony-stimulating Factor (G-CSF)	107 (10)	**204 (25)***
Granulocyte-macrophage Colony-stimulating Factor (GM-CSF)	23 (1.4)	30 (2.0)
Interferon gamma (INF-γ)	3.1 (0.37)	3.4 (0.24)
Keratinocyte Chemoattractant (KC)	225 (31)	**422 (49)***
Monocyte Chemoattractant Protein-1 alpha (MCP-1 α)	12 (1.3)	15 (2.5)
Monocyte Chemoattractant Protein-1 beta (MCP-1 β)	23 (5.2)	20 (3.3)
Regulated And Normal T-cell Expressed and Secreted (RANTES)	4.3 (0.44)	5.5 (0.74)
Tumor Necrosis Factor alpha (TNF-α)	17 (1.4)	24 (2.2)
